# Preliminary Study for Designing a Novel Vein-Visualizing Device

**DOI:** 10.3390/s17020304

**Published:** 2017-02-07

**Authors:** Donghoon Kim, Yujin Kim, Siyeop Yoon, Deukhee Lee

**Affiliations:** 1 Center for Bionics, Korea Institute of Science and Technology, Seoul 02792, Korea; kim.5404@buckeyemail.osu.edu (D.K.); yujin_429@kist.re.kr (Y.K.); h14515@kist.re.kr (S.Y.); 2 Department of Electrical and Computer Engineering, the Ohio State University, Columbus, OH 43210, USA; 3 Department of Biomedical Engineering, Korea University of Science and Technology, Deajeon 305-350, Korea

**Keywords:** venipuncture, vein-visualizing device, penetration, near-infrared light, image processing

## Abstract

Venipuncture is an important health diagnosis process. Although venipuncture is one of the most commonly performed procedures in medical environments, locating the veins of infants, obese, anemic, or colored patients is still an arduous task even for skilled practitioners. To solve this problem, several devices using infrared light have recently become commercially available. However, such devices for venipuncture share a common drawback, especially when visualizing deep veins or veins of a thick part of the body like the *cubital fossa*. This paper proposes a new vein-visualizing device applying a new penetration method using near-infrared (NIR) light. The light module is attached directly on to the declared area of the skin. Then, NIR beam is rayed from two sides of the light module to the vein with a specific angle. This gives a penetration effect. In addition, through an image processing procedure, the vein structure is enhanced to show it more accurately. Through a phantom study, the most effective penetration angle of the NIR module is decided. Additionally, the feasibility of the device is verified through experiments in vivo. The prototype allows us to visualize the vein patterns of thicker body parts, such as arms.

## 1. Introduction

Venipuncture is a process for obtaining blood from a vein, and it is done frequently during medical checks or blood donation. However, subcutaneous fat or dark skin color hinders the visualization of vein structures. Thus, the nurse has no choice but to perform a blind stick based on anatomical knowledge and their experience. These shortcomings leave room for human error, which may lead to direct or indirect harm, including severe cases leading to death [1,2,3]. In this sense, if it is easier to visualize the vein structures on the hand-dorsal or arm, it could be possible not only to prevent the side effects as well as serious direct or indirect risk factors of phlebotomy errors, but also to reduce a significant amount of practice and effort required in phlebotomy.

Fortunately, some devices helping the venipuncture procedure by visualizing vein patterns exist in the market, and many of them uses near-infrared (NIR) light because it has several benefits. Low cost and safety of NIR is the one of the reasons. Two types of infrared light methods are mostly used to visualize veins. One is by using far-infrared (FIR) light. The other one is by using NIR light. By utilizing these two types of IR camera methods, researchers can obtain images of procedures ranging from visualizing vein structures to performing segmentation. For the FIR light method [4], a thermal camera is needed owing to the absorption of water. The FIR method could help one collect high quality images for segmentation, some research has been performed with the FIR method. This method, however, still needs to overcome the disadvantage of high cost. Instead of the expensive FIR method, the NIR light range of the spectrum (700 nm–2500 nm) can be used to visualize the vein pattern under the skin. Although a collected image by using NIR method has low quality, this method is relatively cheaper than the FIR method. In the addition, the safety of NIR is proved [5].

Secondly, ‘NIR window’ exists. Peripheral veins of the arm for venipuncture, are usually located up to a few millimeters below the skin surface. However, melanin and hemoglobin highly absorb the visible range of the spectrum (400 nm–700 nm) [6]. Because of this, the vein structure cannot be observed easily without any supporting devices. However, in the infrared range of the spectrum (700 nm–1 mm), especially above 900 nm, the absorption of water increases, whereas the melanin and hemoglobin levels are lower [5]. Above 1300 nm, water in the skin absorbs all photons [7]. Therefore, the so called ‘NIR window’ is observed in the range of 700 nm~1000 nm. Further, the absorption of blood in the near-infrared window range is sufficiently high to create a large contrast between the vein and surrounding tissue [5]. In this sense, using infrared light can be one of the methods of solving this problem.

**Figure 1:**
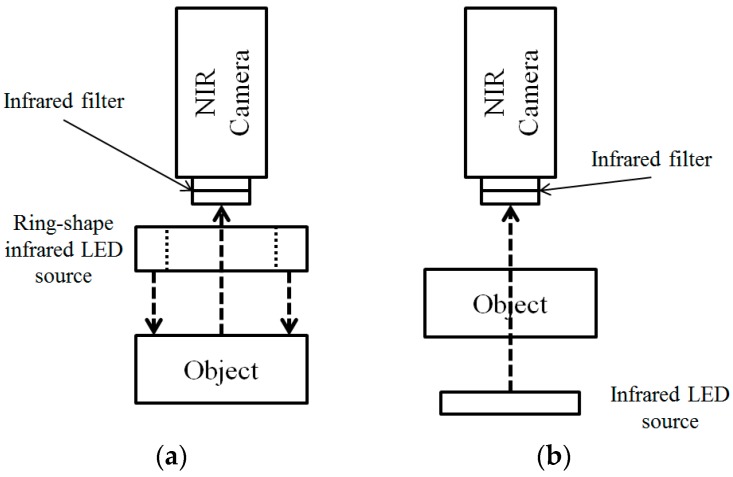
Two types of vein image collection system using the NIR camera: (**a**) Reflection method; (**b**) Penetration method.

For the NIR method, two types of vein image collecting systems are used widely. One is based on reflection and the other one is based on penetration, as shown in Figure 1. As the reflection method is highly affected by reflected lights, the collected image becomes brighter. Thus, it not only has lower contrast but also limited visibility compared to the penetration method. Also, the penetration method also has limitations. To be specific, even though it is applied successfully for detecting vein structures on the hand-dorsal, it cannot penetrate to a sufficient depth in an adult’s arm at a common site for venipuncture.

Many kinds of vein visualizing devices use above methods, reflection or penetration of NIR light. However current devices in the market, have drawbacks. They are big or inconveniently designed. So it is difficult to use that device alone during the venipuncture. Also they have a difficulty when visualizing deep vein. In the addition, the high cost becomes a barrier to access [8]. For instance, Veinviewer (Luminetx, Memphis, TN, USA) projects an image of vessels in green on the skin by using the reflection method, and AccuVein (AccuVein LLC, Cold Spring harbor, NY, USA) also projects a red image with vessels in black on the skin. These types of devices have to be used in darker settings for projection. Furthermore, the projection image can be less accurate depending on the projection angle. Other devices such as the Transilluminator Device (Rabin & Berdo, P.C., Washington, DC, USA), Vein Navigation Device (Novarix Ltd., Abingdon, UK) and VascuLuminator (de Konigh Medical Systems, Arnhem, The Netherlands) use the penetration method. Despite this, they all have their own drawbacks. The Transilluminator Device technique can only be used for veins on the hand-dorsal. VascuLuminator can visualize blood vessels in the hand and wrists of children and adults but is not able to visualize the vein patterns on adults’ arms. In addition, in the case of the Vein Navigation Device, the device captures NIR vein images from the patient’s skin and displays them through a screen. However, as the device and screen visually mask the skin during the process, the intuitiveness and accuracy of the process are significantly impeded.

**Figure 2:**
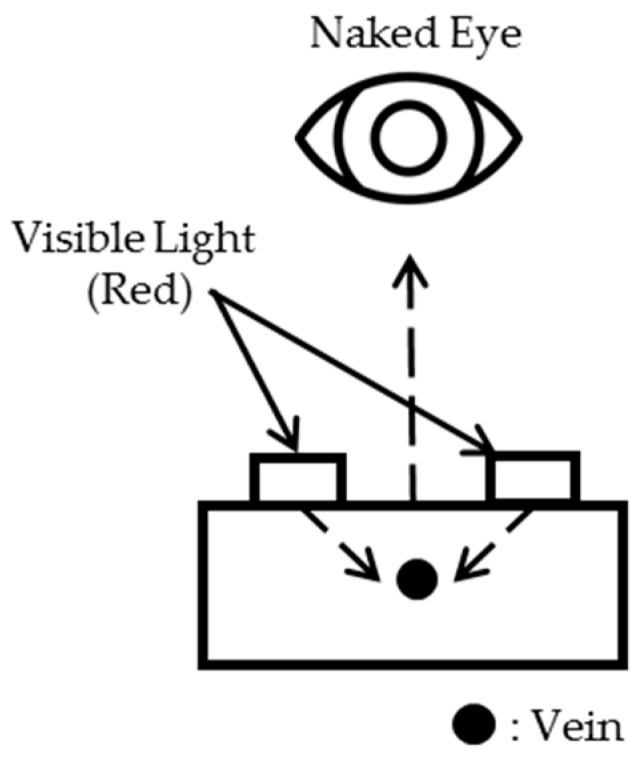
Another penetration method using NIR camera.

Meanwhile, Veinlite (TransLite LLC., Sugarland, TX, USA) , not using an NIR light, use the penetration method but applies in the direction opposite to the penetration at Figure 1, as shown in Figure 2. This is an attempt to avoid the issue that the common penetration method in Figure 1 cannot visualize vein patterns of thicker body parts such as arms. Because the human arm is one of the most common sites of venipuncture and the vessels for venipuncture on the arm are not located at a significant depth, using the direction opposite to that of penetration seems feasible. However, in the first attempt to locate veins of patients, there was at most a 3.2% difference in peripheral intravenous insertion success rates between Veinlite and standard of care [9]. Further, as this device uses a high intensity halogen bulb, it consumes considerable energy [10]. A comparison of three image collecting methods can be checked at the Table 1.

**Figure 3:**
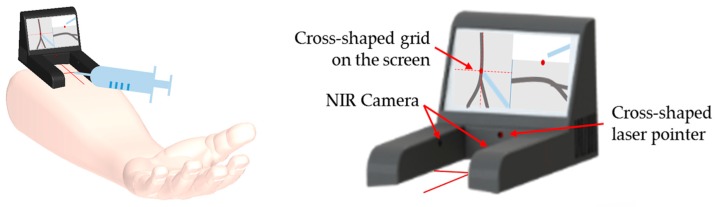
Description of (**left**) usage of proposed device; (**right**) a detail of device.

In this sense, the researchers, Sangjun Lee, Se Hyung Park and Deukhee Lee [11], of the previous study proposed a new vein-visualizing device. As shown in Figure 3, the proposed device will improve the intuitiveness by resolving the visual separation between the two scenes. To be specific, the device projects a cross-shaped laser light onto the skin indicating the puncture point. In addition, the same cross-shaped grid appears on the screen. Through these two identical cross-shapes, the operator can easily match the two visual feedbacks. Furthermore, the proposed device is portable and by based on stereo camera system, it can give a depth information of vein that would be helpful for venipuncture. Also through the simple configuration, it is expected to be enable to lower cost.

They also conducted a phantom study on the propagation of NIR rays under the skin with the IR 695 nm long-pass filter and NIR light diode module, which emits NIR rays of 740 nm. In that study, they showed that the vein model takes much light when the NIR light propagates perpendicularly to the surface just above the vein model. However, the researchers recommended a large incidence angle between the NIR diode module and skin. This is because the NIR module visually occludes the skin in the perpendicular angle [11].

This paper proposes methods for improving and developing the device, inspired by Sangjun Lee et al. [11]. The angle of penetration of method 3 is changed to utilize the advantages of perpendicular propagation and discard the disadvantages of visual hindrances. The remainder of this paper consists of the following sections. Section 2 explains methods for the hardware and image processing. In the next section, various experiments were conducted using phantoms and in vivo. The way of obtaining several parameters that drive the design of the proposed device is presented in Section 3.1. Then, the experimental results in Section 3.2 verify the effectiveness of the proposed device and algorithm. Finally, this paper will be finished with a discussion and problematic points to be solved in future work.

## 2. Methods

Proposed methods are composed of two parts. One is for designing an NIR light module, and the other one is for image processing to enhance the visual effect. First, a limitation of current methods is presented, and then a new model to overcome the drawback is suggested. For the last, an image processing method to show veins more accurately, is explained.

### 2.1. Methods for Equipment

To visualize the vein model and improve the previous module, several pieces of equipment were used such as an NIR CCD camera (Grasshopper3 GS3-U3-41C6NIR-C, Point Grey Inc., Richmond, BC, Canada) and a high resolution lens (GMTHR48014MCN, Goyo Optical Inc., Asaka, Japan). To improve and overcome the shortcomings of the previous study [11], the incidence angle of propagation is adjusted. Although the absorption of deoxyhemoglobin has a peak value near 750 nm, the maximum intensity ratio between the blood vessel and surrounding tissue has a peak value near 850 nm [5,6]. In the case of the vein visualizing process, the advantage of the maximum intensity ratio far outweighs the disadvantage of the absorption of deoxyhemoglobin at 850 nm. Therefore, the vein can be observed well at this wavelength. It is why an 850 nm band-pass filter (BP850-S44.5, Midwest Optical System Inc., Palatine, IL USA) is used instead of a 695 nm long-pass filter. For the same reason, 850 nm emitting diodes are used for the NIR diode module.

In the following sections, first a limitation of the reflection method will be confirmed by comparing with a current penetration method. Second, a drawback of a conventional penetration approach will be explained simply which is presented by Cuper et al. [5]. Then the development of the device and the working principle of the module will be introduced to overcome the limitations.

#### 2.1.1. Limitation of the Reflection Method

**Figure 4:**
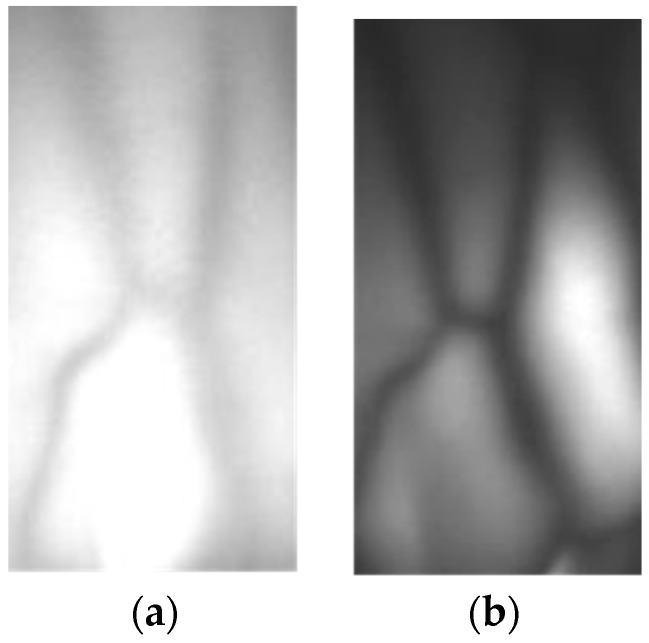
Vein images on hand-dorsal. (**a**) Reflection; (**b**) Penetration.

**Figure 5:**
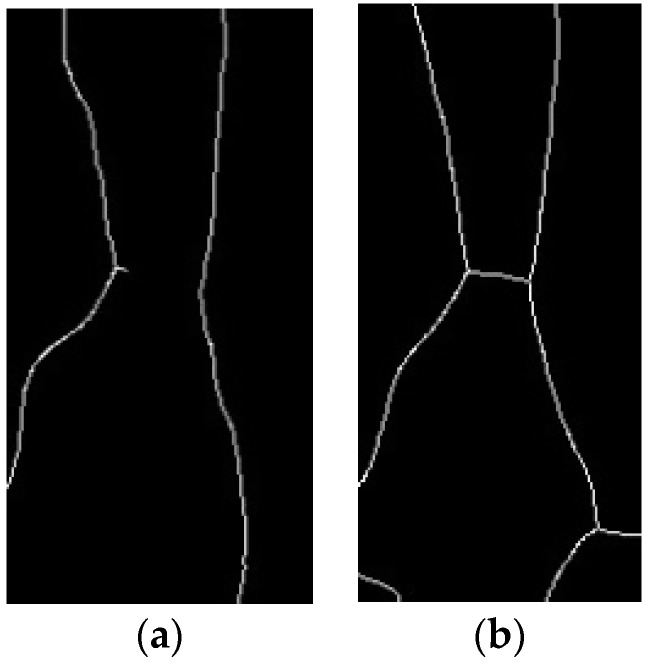
Vein structures on hand-dorsal. (**a**) Reflection; (**b**) Penetration.

To compare the reflection method to the conventional penetration method, an experiment is conducted. Figure 4 shows the images obtained by using method 1 (reflection) and method 2 (penetration) respectively as in Figure 1. To evaluate the effectiveness of each method, vein structures is extracted by skeletonizing the images [12]. The image using the penetration method allowed us to obtain more accurate morphology results as shown in Figure 5. This shows that the existing penetration method is better than the reflection method when using 850 nm NIR light.

#### 2.1.2. Limitation of the Existing Penetration Method

As presented in Section 2.1.1, using an existing penetration method shows better performance when extracting the vein pattern from the images. However, the conventional penetration method also has the drawback of limited maximum visibility depth. Natascha J. Cuper and his colleagues [5] used both NIR light and the penetration method to visualize subsurface blood vessels. Although they successfully visualized blood vessels in the hand-dorsal and wrists of children and adults and at the inside of the elbow in small children, they presented that there were some areas for further development because in thicker body parts like the arm the NIR light cannot reach the other side of the arm through the penetration method. This is because of the fact that all trans-illuminated NIR light will be absorbed before penetrating to the other side of the arm [5]. For this reason, this paper proposes a new penetration method.

#### 2.1.3. Working Principle of the NIR Module

**Figure 6:**
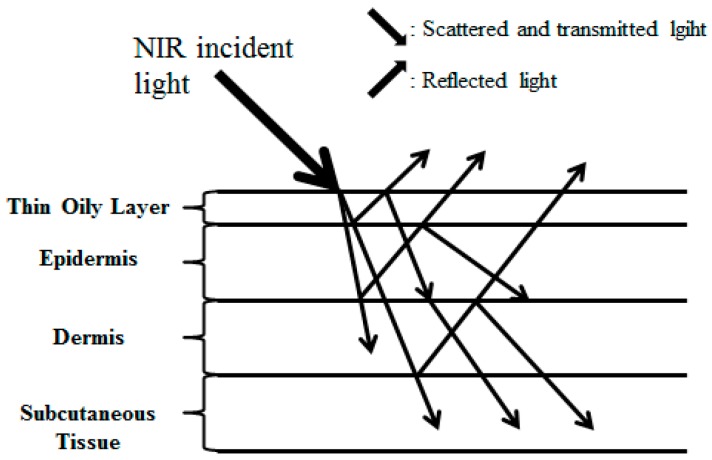
Path of the NIR incident light in skin.

When NIR light penetrates human skin, it is scattered [13]. The scattered NIR light generates both transmitted and reflected light as shown in Figure 6. Thus, through the NIR camera, the reflective image of skin tissue can be detected. This skin optic principle is applied to our new device.

The incident NIR light at a peak wavelength of 850 nm penetrates the skin of the arm in the direction of the vein and scatters. The scattered light travels to the vein and provides a penetration effect. The penetrating NIR light is absorbed in the vein. Finally, through the subsurface scattering and absorption in both the vein and layers of skin, the vein pattern can be obtained.

#### 2.1.4. Development of New NIR Diode Module

**Figure 7:**
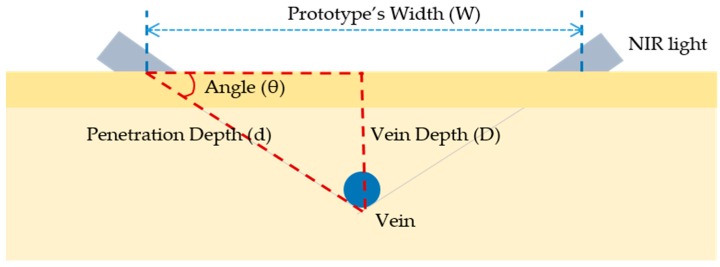
Relation among vein depth (D), prototype’s width (W), and the LED angle (θ).

To overcome the drawback of the conventional penetration method being absorbed before penetrating to the other side of the arm, a new device is proposed in this article. This new and improved device overcomes the limitation to detect vein patterns on arms by penetrating two scenes at the same time from a penetration angle. To be specific, two lines of NIR diodes’ ray penetrates the skin in the middle. If we know the vein depth (D) and penetration depth (d), the penetration angle (θ) could be calculated by a trigonometric relation on Figure 7. Values of the parameters were decided through a phantom study. The process for finding the values will be presented at the Section 3.1.

**Figure 8:**
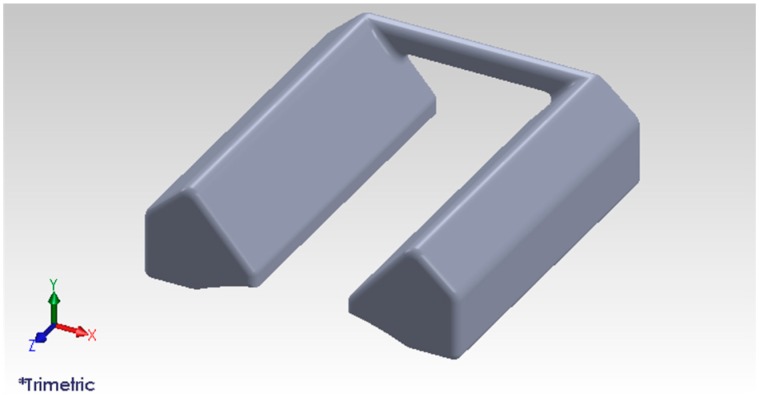
The prototype of the newly proposed device.

The prototype of the device is shown in Figure 8, which was designed by SolidWorks and printed by a 3D printer (Objet260 Connex2 multi-material 3D Printer, Stratasys, Eden Prairie, MN USA). The prototype of the NIR diode module contains two lines of NIR diodes including 20 NIR diodes (850 nm). The NIR CCD camera captures NIR vein images from the skin through the infrared IR filter (850 nm).

### 2.2. Methods for Image Processing

**Figure 9:**
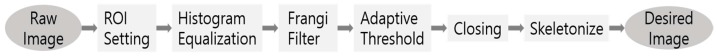
The flow chart of image processing.

The purpose of the newly proposed device is to visualize vein structure in thick body parts, like arms, as shown in Figure 3. Thus, the subsurface veins should become visible on the screen of the device. In other words, since the screen of the device displays the vein structure, image processing is required to extract and show the exact vein pattern from the arm. Figure 9 shows the flow charts for image processing. First, ROI setting method will be presented. Second, a complex histogram equalization method will be introduced that uses both a global histogram equalization image and a contrast-limited adaptive histogram equalization image. Then brief explanation of the multi-scale line enhancement filter (or Frangi filter) will be presented [13]. Then adaptive threshold and morphological closing will be presented. Finally, the desired image with skeleton will be shown. Algorithm is based on OpenCV [14].

#### 2.2.1. Setting the ROI

**Figure 10:**
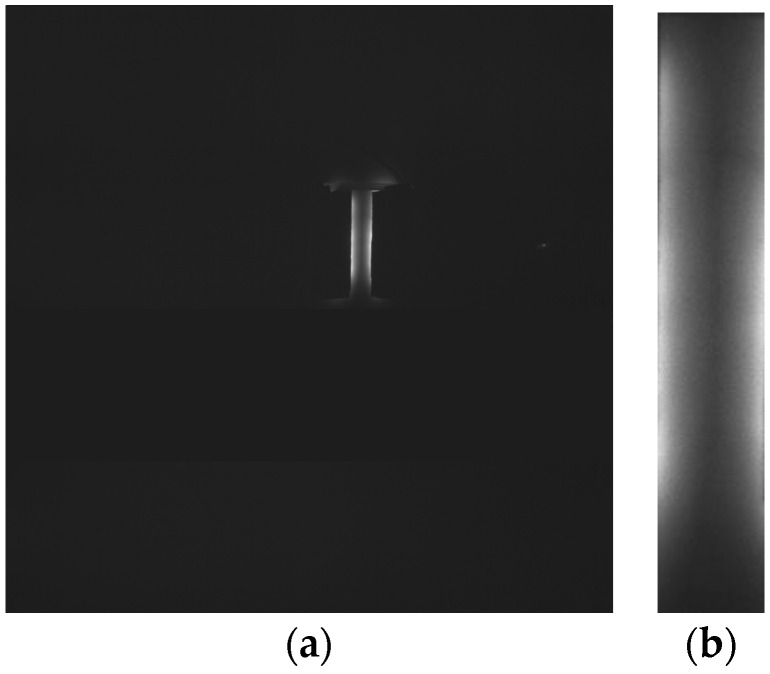
Raw image and ROI image: (**a**) Sample of Raw Image; (**b**) ROI image from (**a**).

ROI (Region of Interest) means the region of an image that one desires to handle. NIR light is shone from both sides of the module, and the band-pass filter creates a dark image except for the ROI, shown as a highlighted segment as in Figure 10a. The ROI image was used only because using the whole image takes too much time and the result would not be optimum. Before the next step, Gaussian blurring was conducted to reduce the noise in the image.

#### 2.2.2. Complex Histogram Equalization

Since the module shines from two sides, non-uniform light distribution exists in the ROI image. This condition makes image processing difficult. To solve this problem, histogram equalization was performed.

Histograms in image processing represent the pixel value distribution of an image. If the image contrast is low, the width of the histogram becomes narrow. Therefore, the histogram is stretched from 0 to 255 to obtain a high contrast image. This procedure is called ‘histogram equalization’.

**Figure 11:**
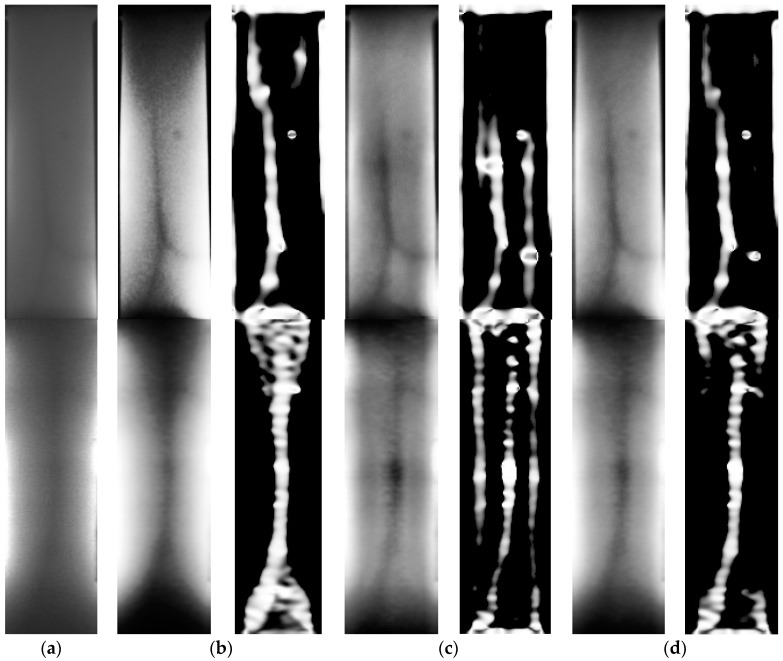
Original image and comparison of different types of histogram equalization and Frangi filter outputs: (**a**) Original image; (**b**) GHE; (**c**) CLAHE; (**d**) CHE.

General histogram equalization (GHE) uses histogram information of the whole image for its transformation function. Though this global approach is suitable for overall enhancement, it works badly with local brightness features [15]. However, contrast limited adaptive histogram equalization (CLAHE) is done locally with small regions of an image. CLAHE reduces the effect of noise by applying the contrast limit. In the case of pixels having values above the contrast limit, they would be clipped before doing the histogram equalization [16]. GHE and CLAHE was compared by applying GHE and CLAHE to the ROI image. In Figure 11, it is possible to observe a reduced effect from the non-uniform light distribution in the image with CLAHE method comparing to the GHE method. Then, a Frangi filter [17] was applied to the output of the histogram equalization. The details of Frangi filter will be explained at the following section. The filter output from the GHE image cannot capture a branch line. However, the output from the CLAHE image can enhance a branch line, but also captures a needless black line in the background.

Therefore, the CHE was introduced, obtained by adding half of the GHE image’s intensity with half of the CLAHE image’s intensity. Through this new CHE method, a better result was observed. It captured the branch lines better than GHE and also suppressed the black line that was not necessary in the background. The comparison result is shown in Figure 11.

#### 2.2.3. Vein Structure Enhancement Method Using 2D Line Filter

Frangi filter or multiscale vessel enhancement filter is used to enhance the *Vesselness* before applying an adaptive threshold to get better segmentation. Frangi et al. proposed a 3D multiscale vessel enhancement filter (or Frangi filter) based on a Hessian matrix (or Hessian) analysis [17]. This enhances a tube-like structure for several scales by considering eigenvalues of the Hessian matrix. For this paper, adjusted method to a 2D image was used. When analyzing a 2D image intensity: *I(x)* where *x* = (*x, y*), it could be considered as Taylor series expansion starting from x_0_.

(1) $$I\left(x\right)≅I\left(x_{0}\right)+\Delta x^{T}\cdot \nabla I\left(x_{0}\right)+\frac{1}{2}\Delta x^{T}H\left(I\left(x_{0}\right)\right)\Delta x,$$

(2) $$\Delta x=x-x_{0},$$

The second term of the right hand side in Equation (1) is the directional derivative of *I*, and the third term represents the second order directional derivative with the Hessian matrix. In the second term, $\nabla I\left(x_{0}\right)$ refers to the image gradient at **x**_0_. Since the image gradient indicates the direction of maximum intensity change, it is orthogonal to the direction of constant intensity. Next, $H\left(I\left(x_{0}\right)\right)$ indicates the Hessian matrix at point **x**_0_. The local shape of *I* is determined by the third term with *H*. Therefore, by getting the eigenvalues (λ_1_, λ_2_) and the eigenvectors (ν_1_, ν_2_) of the Hessian matrix, we can determine the shape and the principal direction of image *I*. Let |λ_1_| ≤ |λ_2_|. Then particularly for the vessel structure, λ_1_ is almost 0 and λ_2_ is large. For the eigenvectors, the direction of ν_1_ is along the vessel, while ν_2_ is orthogonal to ν_1_. Therefore, with this combination of eigenvalues tubular structures can be distinguished. The differentiation is calculated by convolution with a derivative of the Gaussian function. It can be described as

(3) $$\frac{\partial }{\partial x}I\left(x;\;\sigma \right)=\sigma^{\gamma }\frac{\partial }{\partial x}G\left(x;\;\sigma \right)*I\left(x\right),$$

where ***G***(**x**; σ) is a Gaussian function with standard deviation σ,

(4) $$G\left(x;\;\sigma \right)=\frac{1}{2\pi \sigma^{2}}\exp\left(-\frac{{\left|x\right|}^{2}}{2\sigma^{2}}\right),$$

Lindeberg [17] proposed the parameter γ to normalize the derivatives. This normalization is important in comparing the response of differentiations for various scales, because as the scale increases, the intensity and deviation decrease. γ has to be set as 1, if no scale is preferred.

In addition, Frangi et al. introduce a ratio in Equation (5) that helps to distinguish between a line structure and a blob-like pattern.

(5) $$R_{B}=\frac{\lambda ₁}{\lambda ₂},$$

This ratio would be 0 when the *λ*_1_ is near 0 (at the line structure) and would be large at the blob-like pattern. At the ROI image, the vessel structure is darker than the background. Therefore, following Frangi’s approach, the line function is defined as:

(6) $$V_{0}\left(s\right)=\{\begin{array}{cc} \;0 & ,\;if\;\lambda ₂<0\\ \exp\left(-\frac{R_{B}^{2}}{2\beta^{2}}\right)\left(1-\exp\left(-\frac{S^{2}}{2c^{2}}\right)\right) & ,\;else \end{array}$$

(7) $$S=\sqrt{\lambda {₁}^{2}+\lambda {₂}^{2}},$$

where *S* is a norm of the Hessian and *β*, *c* are thresholds controlling the sensitivity. The c depends on the gray scale range of the image. To enhance the brightness of the structure, the condition should be inverted. In Equation (6), $V_{0}\left(s\right)$ represents the different scales, *s*. The result of $V_{0}\left(s\right)$ would be biggest at a specific scale (*s*). Therefore, the final equation is,

(8) $$V_{o}\left(\gamma \right)=\underset{s_{\min}\leq s\leq s_{\max}}{\max}V_{o}\left(s,\gamma \right),$$

Since the Frangi filter is very sensitive to noise, a Gaussian blur was applied to reduce noise. The result of Frangi filtering is shown in Figure 11.

#### 2.2.4. Segmentation Using Adaptive Thresholding and Morphology-Closing

The thresholding function can be defined as Equation (9). In the thresholding function, one sets a value called threshold (T). Then pixels with intensity below T become black (0), and the others above T, become white (255).

(9) $$I\left(x,y\right)=\{\begin{array}{cc} 0 & ,\;if\;I\left(x,y\right)<T\\ 255 & ,\;else \end{array}$$

Adaptive thresholding is a kind of thresholding method, but is a little bit different from the basic one, and more efficient in an image with non-uniform light. For the general threshold, only one threshold value is set for the whole image and adjust it globally. Adaptive thresholding, however, locally computes different threshold values for the small different region. Thus, it covers the non-uniform light condition (providing better results than basic thresholding).

Adaptive thresholding was performed on the filtered output. T value was set as the mean of mask area, and the mask size was set as 63 when the image size was 80 × 320. By this step, the vein structure and background could be separated.

After thresholding, a morphology closing operation was performed with structuring element of ellipse with size 13 × 3. Closing is an operator of mathematical morphology. In addition, it can be done with erosion and dilation as,

(10) A•B=(A⊕B)⊖B,

where A is a binary image (thresholding output) and B is the structuring element. Also ⊕ and ⊖ mean dilation and erosion. With this process, we can fill the hole and connect the broken points. With this effect, better skeletons were obtained from the image.

#### 2.2.5. Skeletonizing the Vein Structure

**Figure 12:**
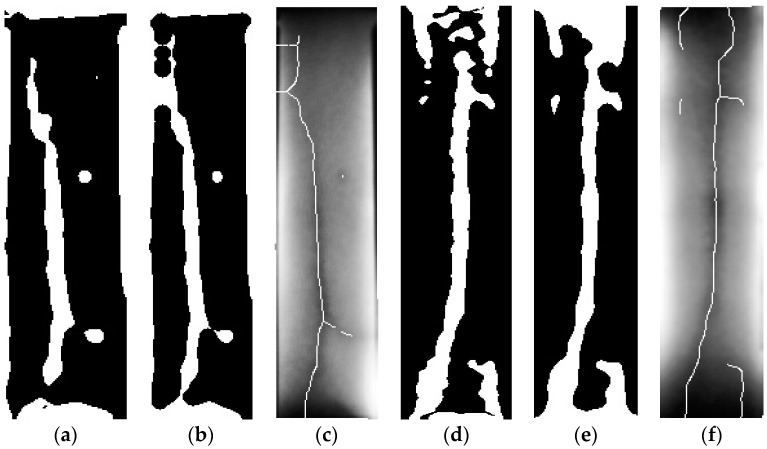
Result of adaptive thresholding, closing output, and desired image: (**a**) Threshold output; (**b**) Closing output of a; (**c**) Desired image of a; (**d**) Threshold output; (**e**) Closing output of d; (**f**) Desired image of d.

A skeleton is a thin centered line, which represents a structure, and ‘Skeletonization’ is a procedure to find a skeleton. To overlay the position of the vein onto the skin more accurately, the image was skeletonized. For this, Zhang-Suen’s thinning algorithm was used [12]. Finally, the image was skeletonized and the skeleton of the vein pattern was found. Then the skeleton image was added to the CHE image to show the vein structure more accurately. The desired image is shown in Figure 12.

## 3. Experimental Results and Analysis

Various experiments were conducted for designing an NIR light module by using a tissue mimicking phantom. Then the feasibility tests were conducted with phantom and in vivo. Finally, the effectiveness of the proposed device was verified through the experiments.

### 3.1. Phantom Study

A phantom study was performed to get some parameters for designing a module, such as a penetration angle and a depth as shown in Figure 7.

First, a tissue phantom was composed based on agar and lipid. An experiment is conducted, to show the effectiveness of an attached penetration mode. Then the penetration depth was measured with the NIR module attached to the skin. The depth, in this paper, was defined as the point at which the intensity becomes 80% of the original intensity. Through that, the NIR light angle could be set. After determining those values, a real module was made and a feasibility test was conducted with a vein model phantom.

#### 3.1.1. Composition of the Tissue Phantom

There are many ways to make a phantom with optical properties similar to tissue. For this phantom study, a solid tissue phantom was made based on a widely used agar and lipid. The composition of the phantom is agar, distilled water, Intra Lipid (IL) and India ink. The ingredients for the phantom are easy to get, not harmful to humans, and are cheaper than other materials [18].

The optical properties of tissue are represented by the scattering ($\mu_{a}$) and absorbance ($\mu_{s}^{\prime }$) coefficient. The role of agar is to make the phantom solid, and it has negligible effects on the performance factors. One can also use gelatin instead of agar, but because the solidity with agar is harder than with gelatin, agar was used instead of gelatin. To meet the desired optical properties of real tissue, IL and India ink were added to the solution. IL is a solution of pure soybean oil and water. It is similar to the actual scattering material in human tissue, so it performs well as the scattering material of the phantom. India ink makes the phantom meet the desired absorbance of the tissue [19,20].

There are multiple steps for making the phantom [18]. First, a pure agar powder is dissolved into the distilled water at about 1% concentration, and melted at 95 °C. Because a heater could burn the agar easily, it should be stirred continuously. After melting the agar, we lowered the temperature to 60 °C while continuously stirring it. At 60 °C, the IL was added as 1 × 10^−2^ concentration and the India ink as 1 × 10^−5^ concentration. Then we lowered the temperature again down to 40 °C while stirring the solution to obtain better uniformity, and poured it into the mold and cooled it at room temperature. Using a refrigerator is not recommended, because it can negatively affect the optical properties of the tissue phantom.

#### 3.1.2. Comparison of Detached and Attached Penetration Modes

**Figure 13:**
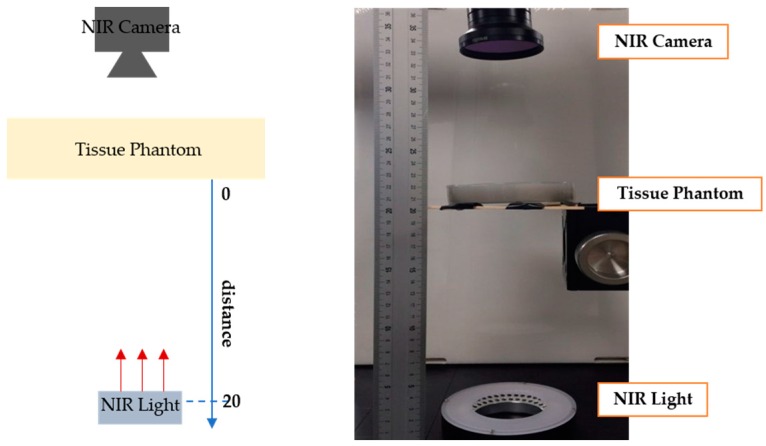
Experimental settings for comparing detached and attached modes: (**left**) description of setting; (**right**) experimental setting.

**Figure 14:**
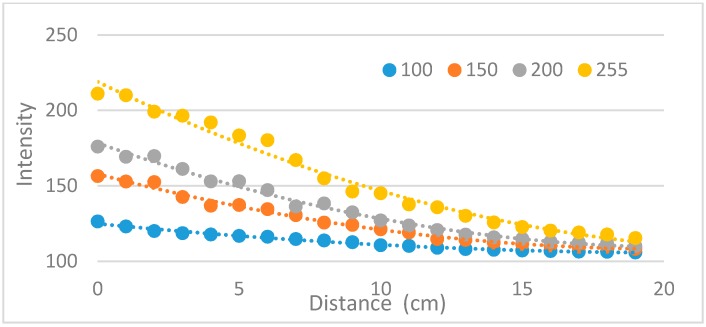
Light intensity penetrated to a tissue phantom along the source to phantom distance changes with different light source intensity.

In this experiment, a phantom with 1.5 cm thickness was made, and NIR camera (Point grey-GrassHopper3 NIR cam) and NIR light were set as shown in Figure 13. Then, the mean intensity of the penetrated NIR light was measured while lifting the NIR light source to the phantom (from distance = 0 to 20 cm). The mean intensity was calculated only using the phantom region. As shown in Figure 14, as the distance between the light source and the phantom decreases, the light intensity becomes stronger. This result can be expected, because brightness is inversely proportional to the square of distance. As expected, the attached mode (distance = 0) shows better penetration than the detached mode (distance = constant).

#### 3.1.3. Measuring the Penetration Depth of the NIR Light Source

**Figure 15:**
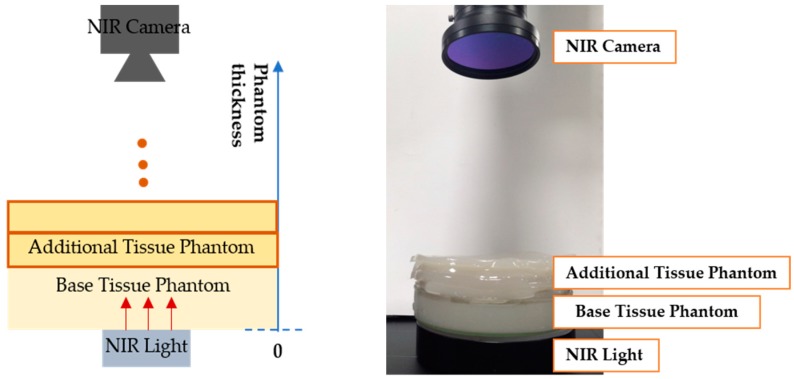
Experimental settings for measuring penetration distance. (**left**) description of setting; (**right**) experimental setting.

**Figure 16:**
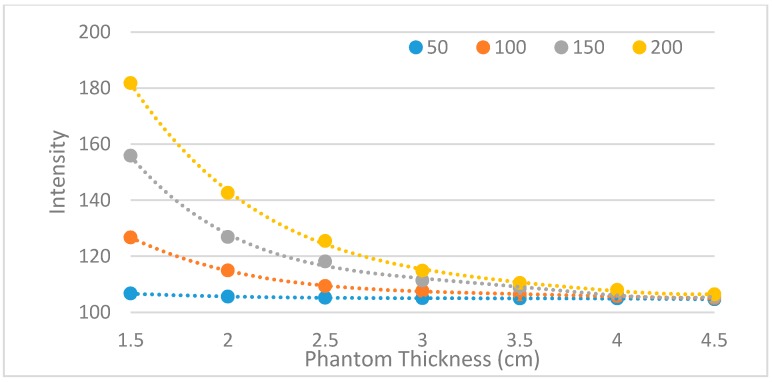
Penetrated NIR light intensity along the phantom thickness at various light source intensities.

As shown in Figure 15, to find the penetration depth of the NIR light source, a base phantom with a 1.5 cm thickness, and additional phantoms with 0.5 cm thickness were made. Then the base phantom was put on the light source and the additional phantoms were added in order. Then the image was captured using the NIR camera. When testing the intensity in a light-off condition, it was 0. As presented in Figure 16, by calculating the phantom part’s mean intensity of images, at about 2 cm thickness, the intensity was around 80% compared to the initial light intensity. As a result, the penetration depth (d) was set as 2 cm.

#### 3.1.4. Determining the LED Penetration Angle of the Prototype

As explained before, a penetration angle was proposed to get a penetration effect by shining the light at the angle. The most commonly done venipuncture site is the *cubital fossa* (same as the *antecubital fossa*), which refers to the part of the arm in front of the elbow. Since there are only small nerves and arteries in this area, there are less pain and less possibility of hematoma [21]. The veins at the *cubital fossa* are usually located about 1.5 cm below the skin [22]. Thus, the mean vein depth (D) was assumed as 1.5 cm. The penetration depth (d) is 2 cm as obtained before. With these values, the effective light propagation angle (θ) could be determined. As shown in Figure 7, the angle could be obtained by Equations (11) and (12):

(11) $$sin\theta =\frac{D}{d},\;D=1.5\;\mathrm{cm},\;d=2\;\mathrm{cm},$$

(12) $$∴\;\theta =sin^{-1}\left(\frac{D}{d}\right)\approx 48.6{}^{\circ}.$$

Through a trigonometric relation, the light width (W) was derived as 2.65 cm. Then a prototype was made using these values.

### 3.2. Feasibility Test

#### 3.2.1. Feasibility Test with a Phantom

**Figure 17:**
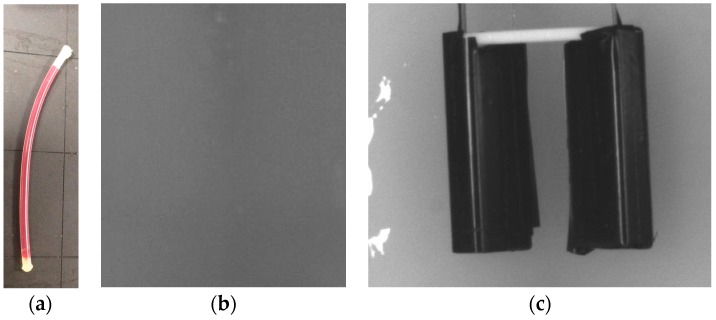
Experimental Settings: (**a**) The vein model; (**b**) The phantom with vein model captured by a camera without NIR light; (**c**) Setting the prototype with the NIR LEDs turned off.

**Figure 18:**
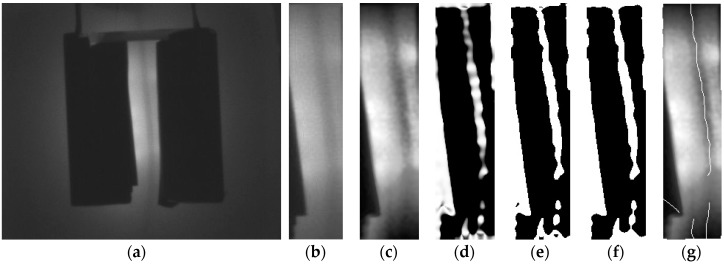
Captured vein model using prototype: (**a**) Raw image; (**b**) ROI; (**c**) CHE; (**d**) Frangi; (**e**) Adaptive threshold; (**f**) Closing; (**g**) Desired image.

A feasibility test was conducted with a phantom. For this experiment, a tissue phantom containing a vein model was made. The vein model is composed of a transparent silicone tube and sheep blood. Since the diameter of a vein at the *cubital fossa* is about 3 mm [22], a silicone tube with a 3 mm diameter was used. Also for the vein model, sheep blood without fibrin, which does not coagulate so could be used for a relatively long time, was injected to the tube using a syringe. Both sides of the tube were then sealed with EVA (Ethylene-vinyl-acetate) glue. Then the tube was located to a 1.5 cm-depth of a mold and the mold was filled with the phantom solution. After the phantom hardened, the phantom was taken out and the experiment was conducted with the designed module. Figure 17c shows the phantom with a prototype. With the prototype on, the vein model was observed more distinctly than with the naked eye as shown in Figure 18. This result illustrates that the proposed device has a feasibility.

#### 3.2.2. Feasibility Test in Vivo

**Figure 19:**
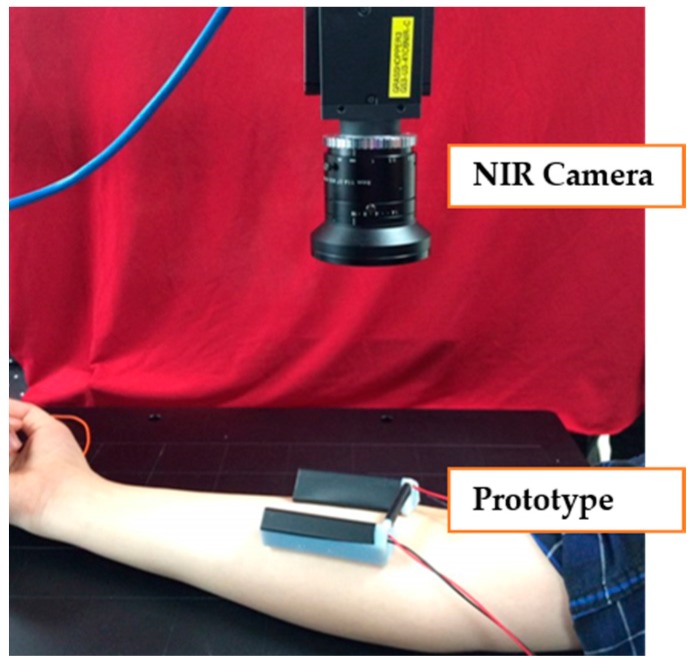
Experiment setting using the prototype.

**Figure 20:**
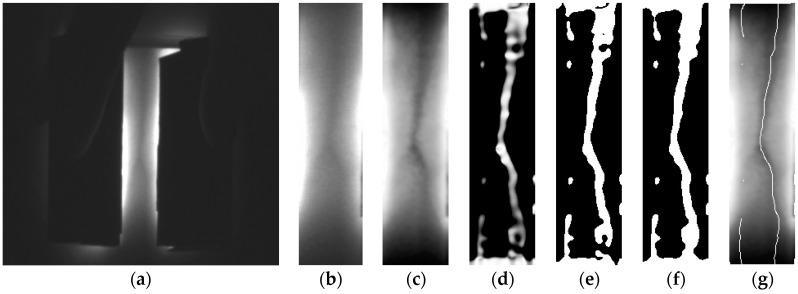
Captured vein model using prototype: (**a**) Raw image; (**b**) ROI; (**c**) CHE; (**d**) Frangi; (**e**) Adaptive threshold; (**f**) Closing; (**g**) Desired image.

As shown in Figure 19, the prototype of the NIR diode module was placed on a subject’s arm and was gently pressed to see the vein more clearly. A raw image was obtained by an NIR camera with an 850 nm band-pass filter. As shown in Figure 20, the veins are clearly visualized in the raw image. As expected from the phantom study, the proposed device also showed good result in vivo.

### 3.3. Verifying the Effectiveness of the Proposed Method

**Figure 21:**
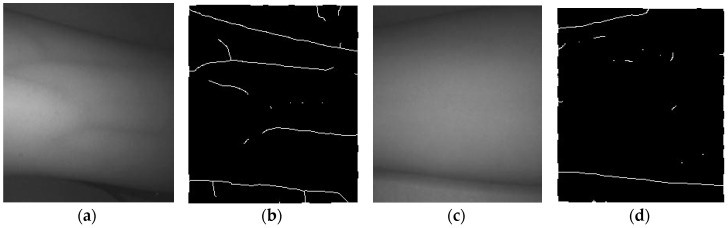
Captured vein images of the arm using the conventional reflection method: (**a**) Patient who has visible veins; (**b**) Skeleton of (**a**); (**c**) Patient who has invisible veins; (**d**) Skeleton of (**c**).

**Figure 22:**
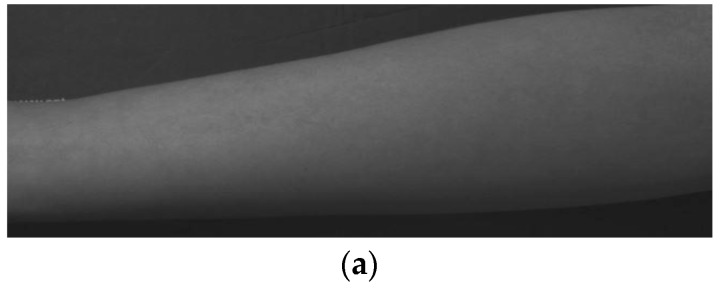

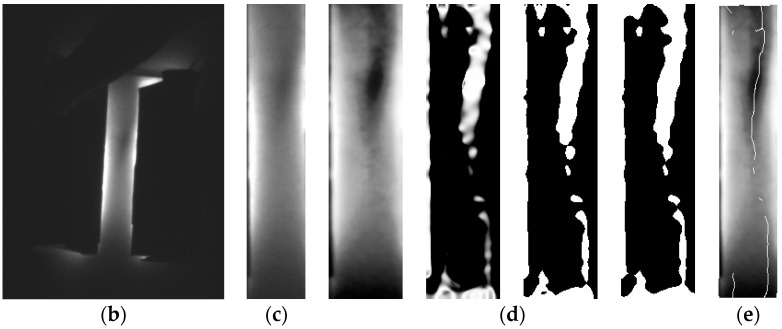
Effectiveness of proposed prototype: (**a**) Arm with invisible vein; (**b**) Arm with prototype; (**c**) ROI image; (**d**) Procedure for getting desired image; (**e**) Desired image.

To evaluate the effectiveness of the proposed device, a reference test was conducted to compare the conventional reflection method to the proposed method. The conventional reflection method in Figure 1 can only be applied to someone whose veins are also visible without any devices. In this case, although the vein pattern looks better than without any supporting devices, as shown in Figure 21a,b, there might not be any significant clinical value for the venipuncture process. In the case of patients whose veins on the arm are not visible, both the NIR light source and the NIR camera cannot visualize the vein pattern, as shown in Figure 21c,d. Since to visualize the vein pattern on the patient of Figure 21c was impossible through the conventional reflection method, the proposed prototype model was tested on this patient. Not only could it visualize a vein pattern at the *cubital fossa* but also skeletonize the vein pattern, as shown in Figure 22.

**Figure 23:**
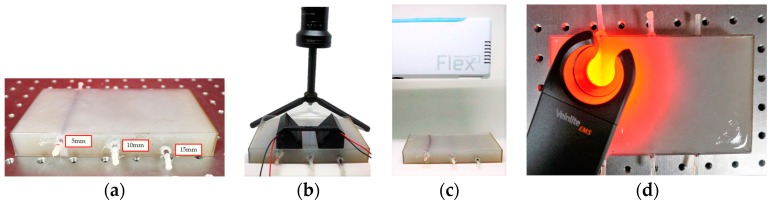
Experimental Setting: (**a**) phantom with vein model; (**b**) Prototype with phantom; (**c**) Veinviewer Flex with phantom; (**d**) Veinlite EMS with phantom.

In the addition, for a comparison with other conventional devices such as Veinviewer Flex and Veinlite EMS, their vein-visualization performances were tested together with the prototype as shown in Figure 23. A tissue phantom, with three 3 mm-diameter vein models located at 5 mm, 10 mm and 15 mm depth respectively, was made for the tests.

**Figure 24:**
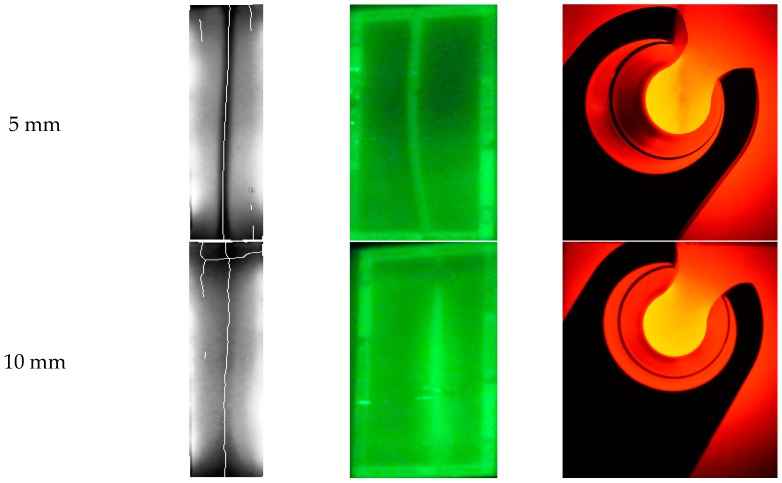

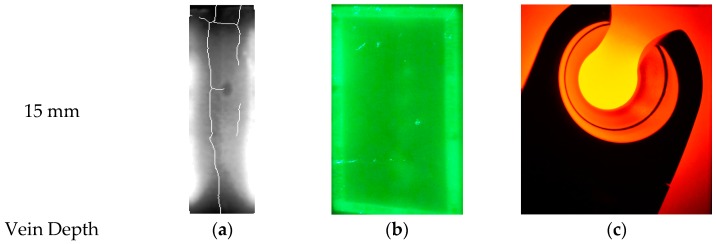
Result of the Experiment: (**a**) Prototype; (**b**) Veinviewer Flex; (**c**) Veinlite EMS.

As shown in Figure 24, the prototype could catch and skeletonize the vein pattern at all depths. But it was effected by a flaw of the phantom. Veinviewer Flex also could visualize the vein pattern at all depths, but not clear at 15 mm depth. Veinlite EMS, however, could only visualize vein at the 5 mm, and showed poor performance at the others. The results show that the prototype has an effectiveness, compared to other conventional devices.

## 4. Discussion

Venipuncture is a very important procedure and is done frequently. However, often a nurse is not able to find a vein because of many hindrances. To overcome these problems, there are many types of vein visualization devices. However, they have difficulty visualizing veins in thicker body parts like an adult’s arm.

In this paper, these drawbacks were investigated and solutions were proposed to counter it. This paper proposed a new design of the NIR light module that has a specific penetration angle. In addition, to show a more accurate line on a screen, an image processing algorithm is proposed. With this design, a penetration effect could applied even to the thicker regions, like an arm. To find the penetration parameter values, a phantom study was conducted. A solid tissue phantom was composed based on agar and lipid, then experiments were performed to get a penetration depth. Through a trigonometric relation, a penetration angle was calculated and the designed module showed good performance within a phantom study and in vivo. After checking the feasibility, additional tests were conducted to verify the prototype. The proposed design made it possible to detect even invisible veins in thick tissue. If a tourniquet is used and arm of a subject is cleaned with the alcohol swab to remove a thin oily layer, the result would be better. In addition, the image processing algorithm effectively enhanced the visual effect. Also when the prototype is compared to the other devices, it showed good performance. Considering the cost of Veinviewer Flex, about $8500, prototype is more competitive. As shown in Figure 3, the proposed device is small and portable. By the simple configuration, the price will be much lowered than the current devices. And through the stereo camera system, it can give a depth information of vein that is necessary for venipuncture. Also by including a screen attached to the NIR diode module, the proposed device does not require any extra skills to match hand-eye coordination. In addition, the device includes a cross-shaped laser pointer. Since the laser pointer is attached and fixed with the NIR module, the indicating point for cannulation does not have any visual distortions deriving from the projection angle. Furthermore, not only are the cross-shaped grid on the screen and the cross-shaped laser pointer fixed with the device but they are also aligned. Therefore, the crossing point can lead the operators to recognize the actual venipuncture point.

The drawbacks are (1) only a rough estimation of the penetration parameters; and (2) undesired skeleton lines on the final image. Since light scatters before it arrives at the vein and the decreasing intensity within the tissue wasn’t considered this research, the designated parameters can still have errors. This can decrease the effectiveness of the proposed device. As shown in the result figures, a needless skeleton line exists, that does not indicate the vein structure. These lines can be confused with the real vein structure. All of these weaknesses will be addressed and improved in future studies.

## Figures and Tables

**Table 1:** Three kinds of image collecting systems.

*Image Collecting System*	Method 1 (Reflection)	Method 2 (Penetration)	Method 3 (Penetration)
***Design***	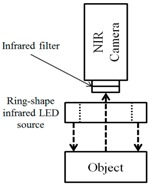	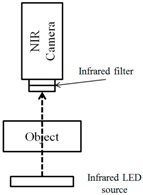	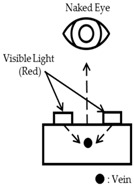
***Characteristic***	Bright image/low contrast, Can’t penetrate thick part like an arm	Can’t penetrate thick part like an arm	No major difference with standard of care/Consumes high energy
***Device***	Veinviewer/AccuVein	Transilluminator Device/Vein Navigation Device/VascuLuminator	Veinlite
